# CD24 Induces Expression of the Oncomir miR-21 via Src, and CD24 and Src Are Both Post-Transcriptionally Downregulated by the Tumor Suppressor miR-34a

**DOI:** 10.1371/journal.pone.0059563

**Published:** 2013-03-22

**Authors:** Santoshi Muppala, Giridhar Mudduluru, Jörg H. Leupold, Daniel Buergy, Jonathan P. Sleeman, Heike Allgayer

**Affiliations:** 1 Department of Experimental Surgery, University of Heidelberg, Mannheim and Molecular Oncology of Solid Tumors, DKFZ, Heidelberg, Germany; 2 Department of Anesthesiology and Intensive Care Medicine, Medical Faculty Mannheim, University of Heidelberg, Mannheim, Germany; 3 Centre for Biomedicine and Medical Technology Mannheim (CBTM), Universitätsmedizin Mannheim, University of Heidelberg, Mannheim, Germany; 4 KIT Karlsruhe Campus Nord, Eggenstein-Leopoldshafen, Germany; University of Barcelona, Spain

## Abstract

Cancer is a complex disease process that evolves as a consequence of multiple malfunctions in key regulatory molecular networks. Understanding these networks will be essential to combat cancer. In this study, we focussed on central players in such networks. In a series of colon and breast cancer cell lines, we found that CD24 activates Src, and induces the activation of c-Jun and expression of c-Jun and c-Fos. Thereby CD24 increases the promoter activity and expression of miR-21, which in turn suppresses expression of Pdcd4 and PTEN. Co-transfection of a CD24 expression construct and an siRNA that silences Src showed that CD24-dependent upregulation of miR-21 is mediated by Src. Additionally, we found that miR-34a post-transcriptionally downregulates CD24 and Src expression, leading to the deactivation of c-Jun, reduced expression of c-Jun and c-Fos, inhibition of miR-21, and upregulation of Pdcd4 and PTEN. Furthermore, miR-34a-mediated inhibition of Src expression reduced migration and invasion of colorectal cancer cells. Resected tumor tissues from 26 colorectal patients showed significantly lower expression of Pdcd4 and miR-34a, and higher expression of CD24, Src and miR-21 compared to the corresponding normal tissues. Moreover, CD24 positively correlated with the amount of Src protein in tumor tissues, and a trend towards an inverse correlation between miR-34a and Src protein levels was also observed. Our results reveal essential players in the complex networks that regulate the progression of solid tumors such as colorectal cancer. These findings therefore identify novel therapeutic approaches for combating tumor growth and progression.

## Introduction

Tumorigenesis is a multistep process that is regulated by complex molecular networks whose activity is perturbed by sequential alterations in a variety of oncogenes, tumor-suppressor genes and microRNA genes [1]. These alterations are usually somatic events, although germ-line mutations can predispose a person to heritable or familial cancer. Subsequent tumor progression ultimately leads to the metastatic spread of tumor cells into distant organs [2], which again is driven by a network of regulatory and effector proteins. Despite many years of basic and clinical research aimed at curbing tumor growth, metastasis remains the prime reason why cancer patients succumb to their disease [3], largely because of the lack of understanding of the complex molecular networks that regulate tumor progression.

Also known as heat stable antigen, CD24 is a glycosylphosphatidylinositol (GPI)-anchored membrane protein that has been implicated in tumorigenesis, progression, metastasis and poor prognosis for a variety of tumor types [4]. Thus expression of CD24 repeatedly emerges from transcrptional profiling as being correlated with tumorigenesis and tumor progression [5], [6]. Functionally CD24 can promote invasiveness and metastasis formation in vivo [7], [8]. CD24 may act in several ways to exert these effects. It can support rolling of tumor cells on endothelial monolayers due to its ability to bind to P-selectin [9], a protein expressed on thrombin-activated platelets [10], [11] and endothelial cells [11], [12]. CD24 also regulates the activity of CXCR4 [13], as well as proliferation, motility and integrin-mediated adhesion [7]. However, much remains to be learned about the activity of CD24 in the context of cancer.

Little is known about the molecular regulatory networks that are addressed by CD24. Our own recent findings suggest that CD24 activates Src within lipid rafts [14]. Src plays a central role in the regulation of invasion and metastasis [15]. Its activity is normally tightly controlled in non-transformed cells, but in may types of cancer, enhanced Src kinase activity is found that correlates with poor prognosis [16], [17]. Activated Src induces AP-1 activation mainly through the MAPK pathway, thus inducing cell migration and invasion [18]. AP-1 family members in turn are key players in multistep tumorigenesis due to their transcriptional activation activities [19].

miRNAs are non-coding RNA molecules that post-transcriptionally regulate gene expression, and can act to either promote or inhibit tumor formation and progression. For example, miR-21 is an oncomir that inhibits the expression of tumor suppressor and/or metastasis suppressor genes such as Pdcd4 and PTEN [20], [21], and is transcriptionally regulated by AP-1 family members [22], [23]. Conversely, miR-34a is a tumor suppressor microRNA that is regulated by the tumor suppressor gene p53 [24], and downregulates expression of tumor progression-associated genes such as Axl and c-Met [25].

In this study we investigated further the molecular pathways addressed by CD24, and thereby have uncovered a regulatory network in which miRNAs play a central role. Specifically we found that CD24-dependent activation of Src increases miR-21 expression, and thereby inhibits expression of Pdcd4 and PTEN. This pathway is counter-regulated miR-34a, which post-transcriptionally inhibits expression of CD24 and Src, resulting in diminished miR-21 expression, and thus enhanced expression of Pdcd4 and PTEN.

## Materials and Methods

### Cell Culture and Antibodies

The human colorectal cancer cell lines (HT-29, HCT-116, Rko, SW480, Colo206f and WiDr) and the human breast cancer cell line MDA-MB-231 were purchased from American Type Culture Collection (ATCC, Manassas, USA), and grown according to the recommended conditions. The human colorectal cancer cell line Geo, a gift from Prof. Douglas Boyd (MD Anderson Cancer Center, Houston, USA), was cultivated in DMEM/10%FCS as published before [20], [25], [26]. Media and FCS were obtained from Invitrogen (Karlsruhe, Germany) and Sigma (Taufkirchen, Germany). Lipofectamine was purchased from Invitrogen (Karlsruhe, Germany), transwell chambers (1 cm^2^/8 mm pore size) were from Costar (Cambridge, USA), and Matrigel was obtained from BD Biosciences (Bedford, USA). Antibodies against Pdcd4 (ab51495) were purchased from Abcam (Cambridge, UK), p-Src-Y416 (#2101), Src-36D10 (#2109), c-Jun-60A8 (#9165) and PTEN-138G6 (#9559)-antibodies were from Cell Signalling (NEB-Frankfurt, Germany), and phospho-c-Jun (sc-822X), c-Fos (sc-52X), anti-IgG control (sc-2338X) and β-actin (sc-1616R) antibodies were from Santa Cruz Biotechnology (Heidelberg, Germany). Mouse monoclonal CD24 antibody (SWA-11) was a kind gift from Prof. Peter Altevogt (Department of Immunology, DKFZ, Heidelberg, Germany) [14], [27].

### Oligonucleotides

Control miR/Scrambled (AM17110), pre-miR-34a (PM-34a) (ID:PM11030), and anti-miR-34a (AM-34a) (ID: AM11030), as well as negative control siRNA (#AM4635), siRNA-CD24 (#ID:s2616) and siRNA-Src (#ID:s13414) were obtained from Ambion (Austin, USA). Taqman primer-probes for the quantification of miR-21 (ID: 000397), miR-34a (ID: 00426), RNU6B (ID: 001093) and Pdcd4 (ID: Hs00377253_m1) were purchased from Applied Biosystems (Foster City, USA), and oligonucleotides for 3′UTR cloning and RT-PCR for CD24 and Src were purchased from Metabion (Martinsried, Germany).

### Expression Plasmids and 3′-UTR-luciferase Reporter Constructs

CD24 cDNA was amplified using the primers indicated in Table S1 and cloned into the pCDNA3.1 vector. The identity of the insert was confirmed by sequencing. The full-length 3′-UTR of CD24 (634 nt) and Src (1814 nt) was amplified using genomic DNA from Geo cells and cloned into the HindIII site of pMIR (Ambion) and the Xba I site of pGL3 (Promega, Madison, WI, USA), respectively. The identity of the insert was confirmed by sequencing. Site–directed mutagenesis (Stratagene, Heidelberg, Germany) to mutate the seed sequences of miR-34a was performed using Luc-CD24-3′-UTR and Src-3′-UTR wild-type sequences as a template. The sequences of cloning primers are provided in Table S1. The constitutively active chicken Src expression plasmid CA10-SrcY527F (A-Src) was used as described previously [14].

### Reporter Assays

Cells were co-transfected in 24 well plates with either 0.5 µg of luciferase construct and pRL-TK (50 ng, Renilla Luciferase; Promega), or together with 50 nM of control miR or PM/AM-miR-34a using lipofectamine 2000. pRL-TK served as an internal control, and its luminescence was measured to normalize transfection efficiency. Dual reporter assays were performed according to the manufacturer’s protocol using the dual-luciferase assay system (Promega). Briefly, 48 h post transfection, cells were washed twice with PBS and lysed with 100 µl passive lysis buffer (Promega) for 20 min, then 20 µl of cell lysate was used for the measurements. Assays for all samples were performed in triplicate, and the results were averaged.

### Preparation of RNA, Protein Extracts, RT-PCR and Immunoblotting

RNA, protein isolation, RT-PCR and western blot analysis were performed as described previously [25]. Expression of CD24 and Src mRNA was determined by SyBr green PCR and actin as a normalizing control (for primer sequences see Table S1). Mature miRNA expression of miR-34a (ID:000426) and miR-21 (ID:000397) were determined by the Taqman miRNA assay (Applied Biosystems, Foster City, CA, USA), and normalized using the 2^−ΔΔCt^ method relative to U6-snRNA (RNU6B; ID:001093 from applied Biosystem, USA). All PCRs were performed in triplicates.

### Chromatin Immunoprecipitaion Assay (ChIP)

ChIP assays were performed as described previously [23]. Briefly, Rko cells were transfected either individually or in combination with the CD24 expression construct, Src siRNA, PM-34a or the A-Src expression construct as indicated. Two days post transfection, the binding of phospho-c-Jun to the miR-21 promoter was measured by RT-PCR with a set of specific primers (sense:5′-GCCTCCCAAGTTTGCTAATG-3′, antisense:5′-TGTACTCTGGTATGGCACAAAGA-3′).

### Cell Migration and Invasion Assay

Rko and HCT-116 cells were transfected with either PM-34a or A-Src or both. Two days post transfection, cells were washed once with ice-cold PBS, trypsinized, counted, and 3×10^5^ cells were seeded on transwell plates either coated with 10 µg matrigel/well (for invasion assays) or uncoated (for *in vitro* migration assays) in serum-free medium containing 0.1% BSA (Bovine serum albumin). As a chemoattractant, 10% FBS in the lower chamber was used. After 14 h, invaded cells were trypsinized and counted using the ATP-luminiscence-based motility-invasion assay (Promega) as previously described [25].

### Tumor Samples

Tissue specimens (tumor, adjacent normal mucosa) from 26 patients with different tumor stages (T1: n = 2; T2: n = 5; T3: n = 16; T4: n = 3) of colorectal cancer were collected after informed written consent from all patients and verification of the samples by a pathologist, and immediately frozen in liquid nitrogen. Tissue screening and documentation process was approved by the institutional “Medizinische Ethik-Kommission II” ethics committee of Medical Faculty Mannheim, University of Heidelberg.

### Statistical Analysis

Statistical analysis was performed using SPSS version 14.0 (SPSS). The Wilcoxon Sign Rank Test was used to compare the expression of Pdcd4, CD24, Src, miR-34a and miR-21 in colorectal tumors and corresponding normal tissues. Spearman correlations among continuous variables were computed. In all tests, p values of ≤0.05 were considered significant and p values >0.05 and <0.1 considered to represent a trend.

## Results

### Ectopic Expression of CD24 Leads to an Increase in the Activity of Src and Induces miR-21 Expression

We recently showed that CD24 interacts with Src and promotes its activity [14]. To investigate further the signalling pathways addressed by CD24 and their possible implications for cancer progression, we first investigated whether ectopic expression of CD24 is able to increase the activity of Src and its downstream signalling axis in a panel of colorectal cancer cell lines. Initially we screened for endogenous expression of CD24 and Src to identify cell lines suitable for loss and gain of function experiments. Thereby Rko, HCT-116, HT-29 and Geo cells were selected as cell lines that express low and high endogenous levels of CD24, respectively, and which differ in their invasive behaviour [25] (Figure S1).

**Figure 1:**
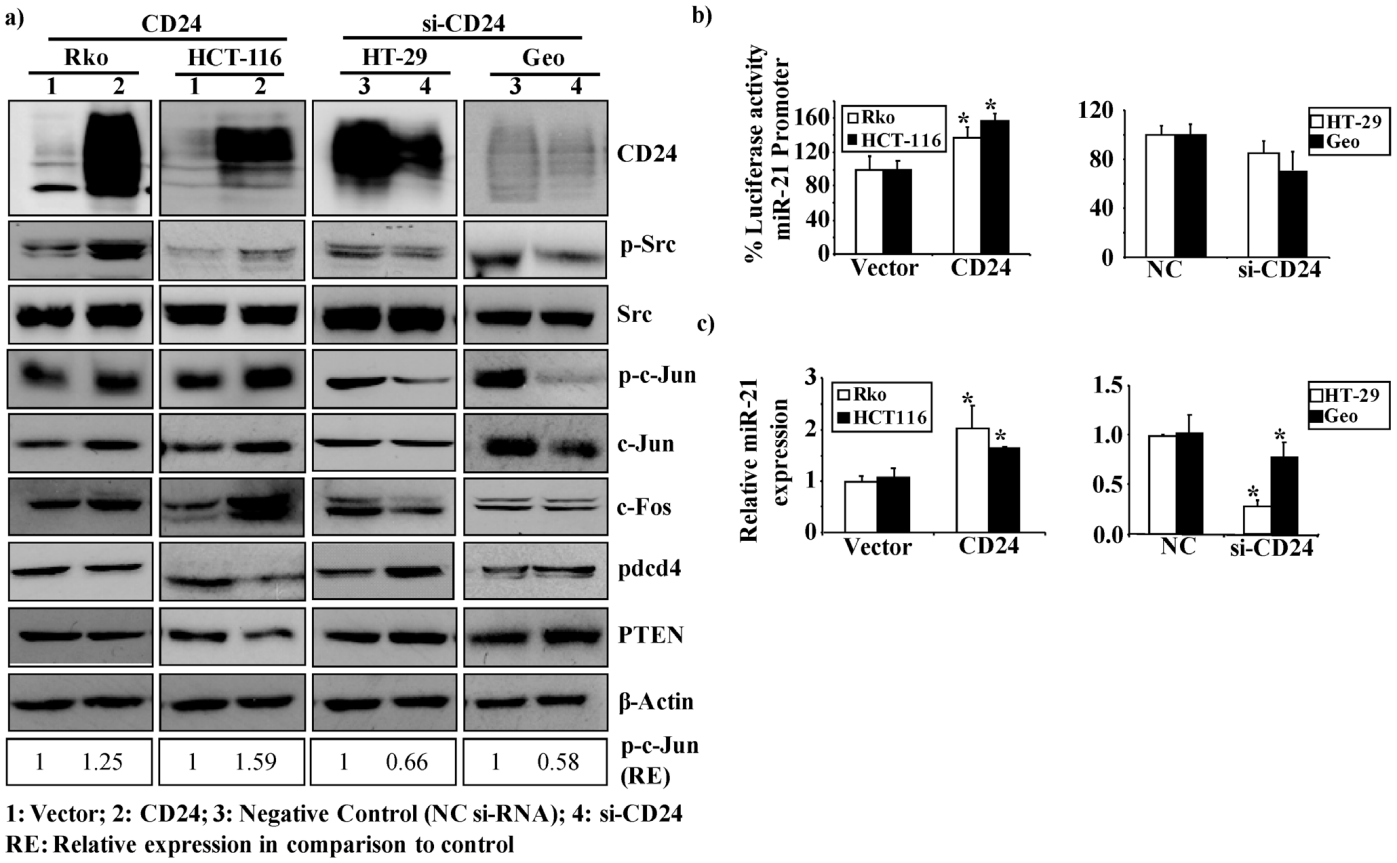
Overexpression of CD24 activates Src and induces miR-21 expression. (**a**) Western blot analysis of CD24, p-Src, Src, phosphorylated c-jun, c-Jun and c-Fos was performed 48 h post transfection. Rko and HCT-116 cells transfected with either with vector control or with a CD24 expression construct is shown in the left panel. Transfection of HT-29 and Geo cell lines either with negative control siRNA (NC) or siRNA against CD24 (si-CD24) is shown in the right panel). β-Actin served as an internal control. (**b**) Luciferase reporter assays of the miR-21 promoter co-transfected either with a CD24 expression construct in Rko and HCT-116 (left panel) or with siRNA against CD24 (si-CD24) in HT29 and Geo cells (right panel) along with respective controls. Percent luciferase activity was calculated either with the miR-21 promoter or control samples set as 100%. The data are presented as the mean ± S.D. Each bar represents the mean value of three biological replicates (Rko: p = 0.01; HCT116: p = 0.02). (**c**) miR-21 expression levels were evaluated by RT-PCR 48 h post transfection upon overexpression or knock-down of CD24 in Rko, HCT-116 or HT29, Geo cell lines, respectively. The data are presented as the mean ± S.D. Each bar represents the mean value of three biological replicates (Rko: p = 0.009; HCT116: p = 0.01; HT29: p<0.001; Geo: p = 0.02). Specific p-c-Jun band intensities were normalized relative to β-actin and are represented as fold change in comparison to the control.

In a first approach, low CD24-expressing Rko and HCT-116 cells were transiently transfected with an expression plasmid for CD24, and the high CD24-expressing HT-29 and Geo cells were transiently transfected with a specific siRNA that targets CD24 (Figure S2a). Ectopic expression of CD24 enhanced the phosphorylation status of Src, and conversely, knock down of CD24 decreased Src phosphorylation (Figure 1a). Next, we investigated the phosphorylation of c-Jun under these conditions, since it is known that Src is able to activate AP-1 transcription factors [18]. Accordingly, we found that ectopic expression of CD24 induced the phosphorylation of c-Jun, paralleled by an increased expression of endogenous c-Jun and c-Fos protein (Figure 1a left panel). The opposite effect was observed upon knock-down of CD24 (Figure 1a right panel). In addition, we screened for miR-21 expression following CD24-overexpression or knockdown, because we and others have shown that miR-21 is regulated by AP-1 family members [22], [23]. To this end, we investigated the activity of the miR-21 promoter in luciferase reporter assays, together with the expression of this micro RNA. Ectopic expression of CD24 significantly induced miR-21 promoter activity as compared to the vector control, and also significantly increased miR-21 expression. Consistently, silencing of CD24 expression decreased miR-21 promoter activity and expression (Figure 1b,c).

**Figure 2:**
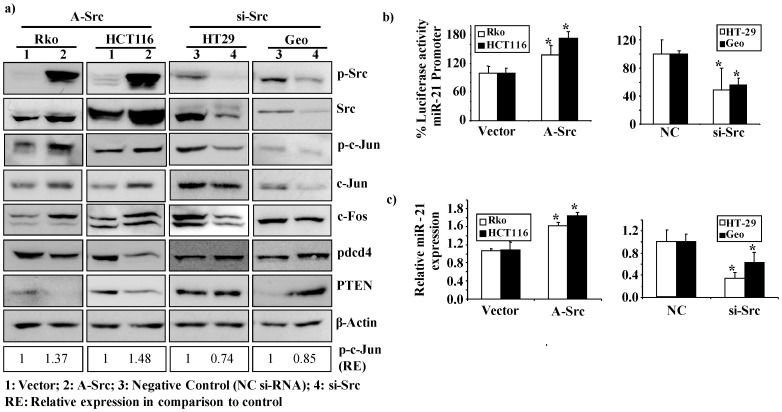
Activated Src induces miR-21 expression. (**a**) Western blot analysis of phosphorylated Src, Src, phosphorylated c-jun, c-Jun, c-Fos, was performed 48 h post transfection. Transfection of Rko and HCT-116 cells either with vector control or with a constitutively active Src expression construct (A-Src) is shown in the left panel. Transfection of HT-29 and Geo cell lines with either negative control siRNA (NC) or an siRNA against Src (si-Src) is shown in the right panel. β-Actin served as an internal control. (**b**) Luciferase reporter assays of the miR-21 promoter co-transfected either with A-Src in Rko and HCT-116 cells (left panel) or with si-Src in HT29 and Geo cells (right panel) along with respective controls. Percent luciferase activity was calculated either with the miR-21 promoter or control samples set as 100%. The data are presented as the mean ± S.D. Each bar represents the mean value of three biological replicates (Rko: p = 0.05; HCT116: p = 0.005; HT29: p = 0.001; Geo: p<0.001). (**c**) miR-21 expression levels were evaluated by RT-PCR 48 h post transfection with A-Src in Rko and HCT-116 cells, or with si-Src in HT29 and Geo cells. The data are presented as the mean ± S.D. Each bar represents the mean value of three biological replicates (Rko: p = 0.05; HCT116: p = 0.005; HT29: p = 0.02; Geo: p = 0.02). Specific p-c-Jun band intensities were normalized relative to β-actin and are represented as fold change in comparison to the control.

In a second approach, we investigated the role of Src in the CD24-mediated induction of miR-21 expression. Consistent with our previous observations, overexpression of a constitutively activated Src in Rko and HCT-116 cell lines (Figure S2b) resulted in an increased phosphorylation of c-Jun and elevated levels of c-Jun and c-Fos protein (Figure 2a, left panel), whereas siRNA-mediated silencing of Src in the HT-29 and Geo cell lines led to the converse effect (Figure 2a, right panel and Figure S2b). Importantly, we found that Src activity is necessary and sufficient for miR-21 promoter activity and expression, as evidenced by ectopic expression of constitutively activated Src in Rko and HCT-116 cells, and specific silencing of Src in HT-29 and Geo cells (Figure 2b, c).

Induction of miR-21 was first observed 48 h after ectopic expression of CD24 or Src, and not at 6 h or 24 h post-transfection. Furthermore, equivalent levels of miR-21 expression were observed at 48 h and 72 h post-transfection (data not shown). In further control experiments, we used additional independent CD24, Src and negative control siRNAs. The results obtain were equivalent to those described above (data not shown), ruling out off-target or other non-specific effects.

**Figure 3:**
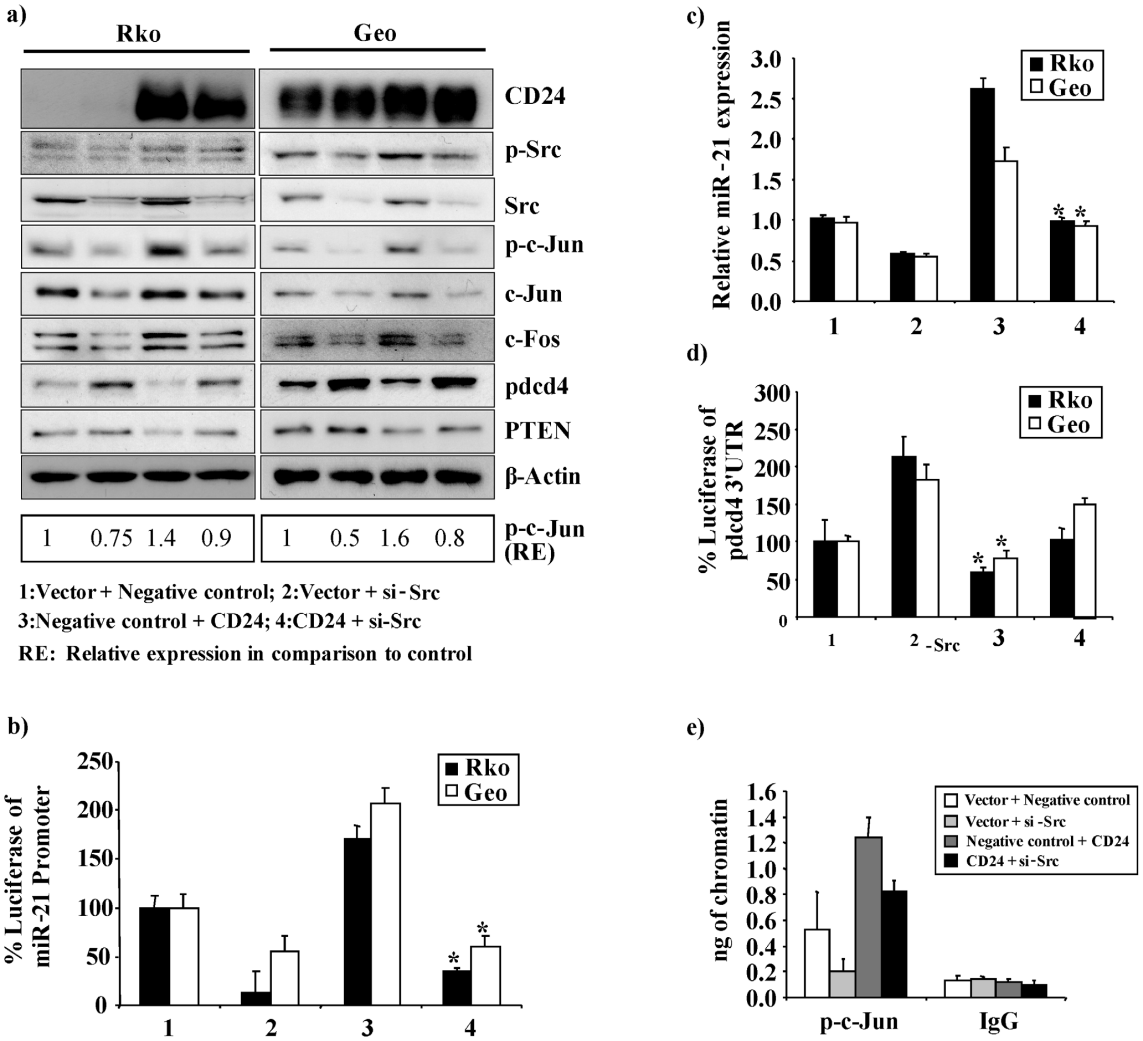
CD24 induced miR-21 regulation is mediated through Src. (**a**) Western blot analysis of CD24, phosphorylated Src (p-Src), Src, phosphorylated c-jun (p-c-jun), c-Jun, c-Fos, Pdcd4 and PTEN was performed 48 h post transfection. Rko (left panel) and Geo cells (right panel) were transiently transfected with either an expression construct for CD24 (CD24), the empty vector (Vector), an siRNA against Src (si-Src) or a negative control siRNA (NC) as indicated. β-actin served as an internal control. (**b**) Luciferase reporter assays in Rko and Geo cells of the miR-21 promoter co-transfected with either an expression construct for CD24 (CD24), the empty vector (Vector), an siRNA against Src (si-Src) or a negative control siRNA (NC) as indicated. Percent luciferase activity was calculated either with the miR-21 promoter or control samples set as 100%. The data are presented as the mean ± S.D. Each bar represents the mean value of three biological replicates. (**c**) miR-21 expression levels were evaluated by RT-PCR 48 h post transfection with either an expression construct for CD24 (CD24), the empty vector (Vector), an siRNA against Src (si-Src) or a negative control siRNA (NC) as indicated. The data are presented as the mean ± S.D. Each bar represents the mean value of three biological replicates. (**d**) Luciferase reporter assays of the Pdcd4-3′-UTR co-transfected with either an expression construct for CD24 (CD24), the empty vector (Vector), an siRNA against Src (si-Src) or a negative control siRNA (NC) as indicated. Percent luciferase activity was calculated either with the Pdcd4-3′-UTR or control samples set as 100%. The data are presented as the mean ± S.D. Each bar represents the mean value of three biological replicates. (**e**) The *in vivo* association of phosphorylated c-jun with the miR-21 promoter was evaluated with a ChIP assay in Rko cells after 48 h of transfection with either an expression construct for CD24 (CD24), the empty vector (Vector), an siRNA against Src (si-Src) or a negative control siRNA (NC) as indicated. DNA immunoprecipitated with the p-c-jun antibody or an isotype IgG control antibody was amplified by real time PCR. Specific p-c-Jun band intensities were normalized relative to β-actin and are represented as fold change in comparison to the control.

To determine whether CD24 is able to induce miR-21 expression specifically through the activation of Src, we ectopically expressed CD24 and silenced Src in Rko and Geo cells, either alone or in combination. As expected, when Src was silenced, diminished phosphorylation of c-Jun compared with the control was observed, which was paralleled by decreased expression of c-Jun and c-Fos proteins. (Figure 3a). In contrast, ectopic expression of CD24 resulted in enhanced phosphorylation of Src and c-Jun, and elevated c-Jun and c-Fos protein expression (Figure 3a, Figure S3). However, when Src was silenced in cells ectopically expressing CD24, phosphorylation of c-Jun and the expression of c-Jun and c-Fos was significantly reduced compared to ectopic expression of CD24 alone (Figure 3a). Additionally, we also observed a significant reduction in miR-21 promoter activity and expression following combined ectopic CD24 expression and Src silencing (p = 0.004) (Figure 3b, c). Similar statistically significant results were observed for the activity of a 4XAP-1 Luc reporter construct under similar conditions (Figure S3). Taken together, these results suggest that the CD24/Src signalling axis phosphorylates c-Jun and induces the expression of c-Jun and c-Fos, resulting in the induction of miR-21 expression.

### CD24/Src-induced miR-21 Expression Downregulates Pdcd4 and PTEN

We and others have found that upregulation of miR-21 is associated with the downregulation of the tumor suppressor genes Pdcd4 and PTEN [20], [21]. To investigate whether CD24-mediated Src activation is able to induce miR-21 and thereby post-transcriptionally downregulate Pdcd4, we investigated the Pdcd4 3′-UTR activity after silencing of Src and/or ectopic expression of CD24. After Src silencing, we found a significant increase in Pdcd4 3′-UTR activity (Rko: p = 0.02; Geo: p = 0.01). Accordingly, ectopic expression of CD24 significantly reduced the activity of the Pdcd4 3′-UTR (Rko: p = 0.03; Geo: p = 0.01) (Figure 3d). Interestingly, we found only a slight induction of 3′-UTR activity when we concomitantly knocked down Src and ectopically expressed CD24 (Figure 3d).

To determine whether AP-1 transcription factors bind to the miR-21 promoter following CD24/Src signalling, ChIP experiments were performed using cells transfected with the specific siRNA against Src and/or the CD24 alone expression construct. Silencing of Src reduced binding of phosphorylated c-Jun to the miR-21 promoter as compared to the control. In contrast, in cells overexpressing CD24 we found an induced binding of phosphorylated c-jun, and a slightly induced phosphorylated c-jun binding in combination of both CD24 and si-Src (Figure 3e). Finally, we assessed the protein expression of Pdcd4 and PTEN [20], [21] and found that CD24-induced expression of miR-21 is able to downregulate the expression of both tumor suppressor genes (Figure 3a). These experiments further support the notion that CD24 is mediating miR-21 expression, and as a consequence suppresses expression of Pdcd4 and PTEN. However, in addition to post-transcriptional regulation of PDCD4 and PTEN by CD24, other mechanisms such as protein degradation/stabilization could also contribute to the protein expression levels of these genes.

### The CD24 and Src 3′-UTRs are Targets of miR-34a

**Figure 4:**
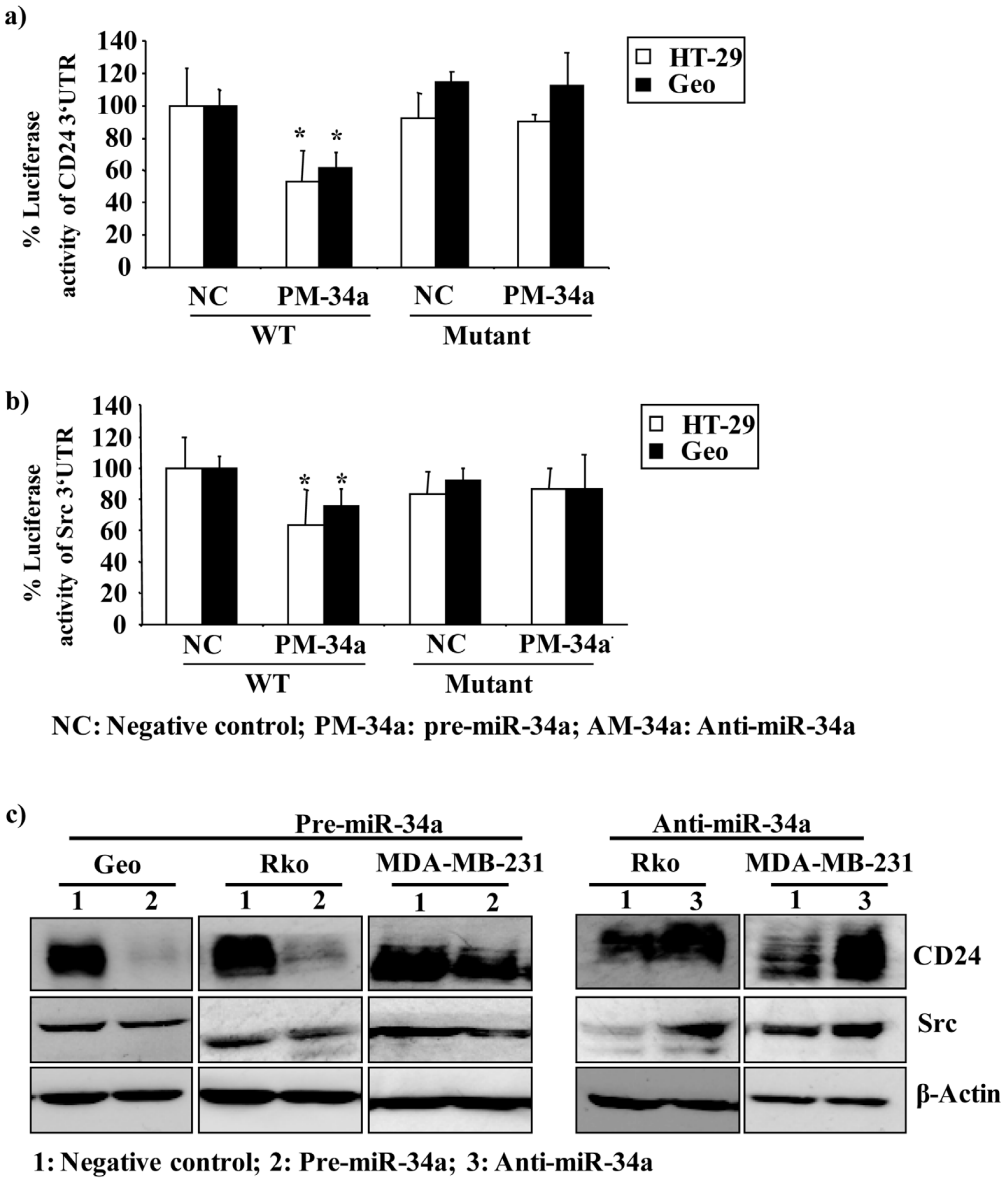
miR-34a targets the CD24- and Src-3′-UTR and regulates their expression. (**a,b**) Luciferase assays using the CD24− and Src-3′-UTR or mutant reporter constructs transfected into HT-29 and Geo cells together with either control-miRNA (NC) or PM-34a. Percent luciferase activity was calculated either with the CD24− or Src-3′-UTR or control-miR samples set as 100%. The data are presented as the mean ± S.D. Each bar represents the mean value of three technical replicates. (**c**) Geo, Rko, HT-29 and MDA-MB-231 cells were transfected either with control miRNA or PM-34a, and 48 h later protein was isolated and western blot analysis for CD24 and Src was performed (left panel). Rko and MDA-MB-231 cells were transfected either with control miRNA or AM-34a, and 48 h later protein was isolated and western blot analysis for CD24 and Src was performed (right panel).

Using *in silico* analysis (miRanda) we found two miR-34a seed sequences in the CD24 and Src 3′UTR. Of these, the seed sequence that showed the higher mirSVR score was used for further analysis. We also found a conserved target sequence for miR-34a in the 3′-UTR of Src. These data suggested that CD24 and Src might be targets of miR-34a. To investigate this hypothesis we first determined the expression of endogenous miR-34a in different colorectal cell lines and found low endogenous expression in HT-29 and Geo cells (Figure S1c, top panel). We then asked whether the 3′-UTRs of CD24 and Src are functional targets of miR-34a in these cells. To this end, we generated luciferase reporter plasmids driven by the SV40 basal promoter, and harbouring either the 3′-UTR of CD24 or the 3′-UTR of Src. These constructs were individually co-transfected together with pre-miR-34a (PM-34a) into the HT-29 and Geo cell lines. Ectopic expression of miR-34a significantly reduced luciferase activity for both the CD24 and Src 3′-UTRs compared to appropriate controls was observed (CD24 3′-UTR: HT-29:p = 0.05 and Geo: p = 0.02; Src 3′-UTR: HT-29: p = 0.028; Geo: p = 0.01) (Figure 4a,b). To verify the miR-34a-mediated regulation of the 3′UTRs of CD24 and Src, luciferase assay experiments were performed with co-transfection of either the Src or CD24 3′UTR reporters along with various concentrations of miR-34a. CD24 and Src 3′UTR activity was reduced in a dose dependent manner (p≤0.05) in response to miR-34a (Figure S5).

To demonstrate the specificity of miR-34a in repressing the CD24 and Src 3′-UTRs, we cloned CD24 and Src 3′-UTR reporter constructs in which the conserved miR-34a seed sequences were mutated for miR-34a interaction (see Figure S4). These two constructs were then co-transfected with or without PM-34a into HT-29 and Geo cells. Neither mutated construct showed any significant change in luciferase activity when compared with the negative control (Figure 4a,b), confirming the specificity of miR-34a. Furthermore, transfection of Geo, Rko, and MDA-MB-231 cells with control miR, PM-34a or AM-34a resulted in the expected inhibition or induction of CD24 and Src protein expression in all cell lines (Figure 4c). In addition, Geo cells were transfected with PM-34a, and the resulting expression of CD24 and Src mRNA was examined. CD24 and Src mRNA expression was reduced significantly by PM-34a (Figure S6). Taken together, our data suggest that miR-34a is a post-transcriptional regulator of CD24 and Src.

### miR-34a Downregulates miR-21 by Targeting CD24 and Src Post-transcriptionally

**Figure 5:**
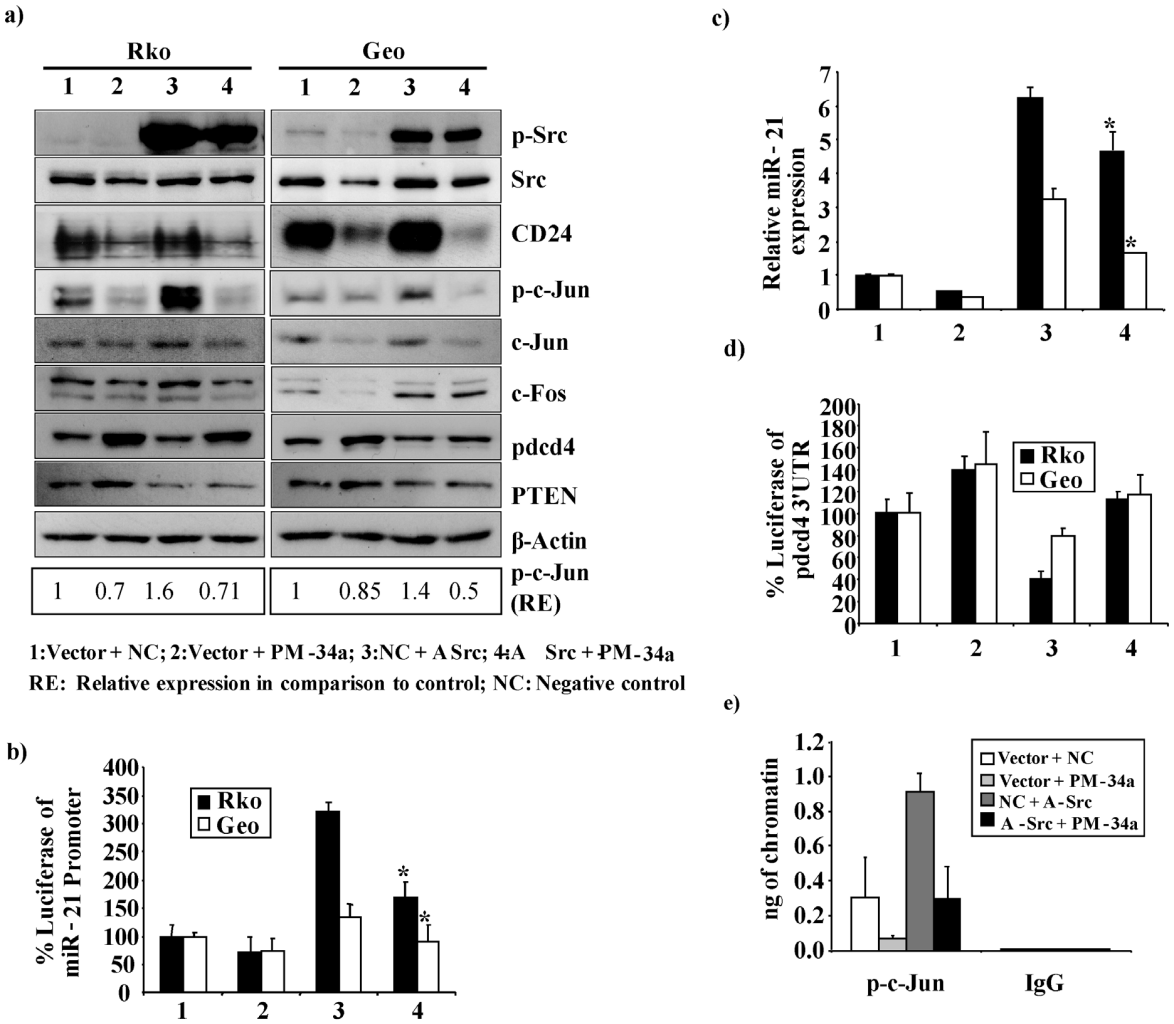
miR-34a downregulates miR-21 by targeting the CD24/Src mediated pathway. (**a**) Western blot analysis of phosphorylated Src (p-Src), Src, CD24, phosphorylated c-jun (p-c-jun), c-Jun, c-Fos, Pdcd4 and PTEN was performed 48 h post transfection. Rko (left panel) and Geo cells (right panel) were transfected with a constitutively active Src expression construct (A-Src), empty vector (Vector), PM-34a or negative control (NC) as indicated. β-actin served as an internal control. (**b**) Luciferase reporter assays in Rko and Geo cells of the miR-21 promoter co-transfected with a constitutively active Src expression construct (A-Src), empty vector (Vector), PM-34a or negative control (NC) as indicated. Percent luciferase activity was calculated either with the miR-21 promoter or control samples set as 100%. The data are presented as the mean ± S.D. Each bar represents the mean value of three biological replicates. (**c**) miR-21 expression levels were evaluated by RT-PCR 48 h post transfection with a constitutively active Src expression construct (A-Src), empty vector (Vector), PM-34a or negative control (NC) as indicated. The data are presented as the mean ± S.D. Each bar represents the mean value of three biological replicates (p = <0.05). (**d**) Luciferase assays in Rko and Geo cells transfected with the Pdcd4-3′-UTR reporter construct together with a constitutively active Src expression construct (A-Src), empty vector (Vector), PM-34a or negative control (NC) as indicated. Percent luciferase activity was calculated either with the Pdcd4-3′-UTR or control samples set as 100%. The data are presented as the mean ± S.D. Each bar represents the mean value of three biological replicates. (**e**) The *in vivo* association of phosphorylated c-jun with the miR-21 promoter was evaluated with a ChIP assay in Rko cells after 48 h of transfection with a constitutively active Src expression construct (A-Src), empty vector (Vector), PM-34a or negative control (NC) as indicated. DNA immunoprecipitated with the p-c-jun antibody or an isotype IgG control antibody was amplified by real time PCR. Specific p-c-Jun band intensities were normalized relative to β-actin and are represented as fold change in comparison to the control.

To investigate whether the tumor suppressor miR-34a can regulate miR-21 through CD24/Src signalling, and to elucidate a possible role for AP-1 family members in such a regulation, co-transfection experiments were performed either with PM-34a, an expression construct for constitutively active Src (A-Src), or with a combination of both. PM-34a inhibited the luciferase activity of the miR-21 promoter and also significantly reduced A-Src-induced luciferase activity of this promoter (Figure 5b; Rko: p = 0.04; Geo: p = 0.02). These findings encouraged us to investigate whether miR-34a inhibits CD24/Src-mediated AP-1 activation and miR-21 regulation. We observed that expression of the c-Jun and c-Fos genes were significantly downregulated at the mRNA level after PM-34a transfection. Furthermore, this treatment abolished A-Src-induced expression of c-Jun and c-Fos mRNA (Figure S7). In contrast, ectopic expression of PM-34a resulted in upregulation of Pdcd4 mRNA, whereas the ectopic expression of constitutively active Src reduced mRNA expression of this protein (Figure S7). Under the same conditions, we performed luciferase reporter assays using the miR-21 promoter (Figure 5b), a 4XAP-1 Luc reporter construct as a positive control for AP-1 transcriptional activity (Figure S6), and the Pdcd4 3′-UTR (Figure 5d), as well as quantitative PCR for miR-21 (Figure 5c), ChIP analysis for phosphorylated c-jun binding to the miR-21 promoter (Figure 5e) and Western blot analysis for CD24, Src, phosphorylated Src, phosphorylated c-jun, c-Jun, c-Fos, Pdcd4 and PTEN (Figure 5a). Consistent with our previous observations, transfection with PM-34a reduced CD24, Src, phosphorylated c-jun, c-Jun and c-Fos protein expression. Furthermore, diminished miR-21-promoter activity and expression was observed, which was accompanied by increased expression of the Pdcd4 and PTEN proteins (Figure 5). Moreover, ChIP demonstrated inhibition of the binding of phosphorylated c-jun to the miR-21 promoter after pre-miR34a treatment *in vivo* (Figure 5e). Taken together, these data suggest that miR-34a downregulates miR-21 expression by targeting the CD24 and Src 3′-UTRs.

For additional controls, we investigated the role of miR-34 in the regulation of the expression of other miRNAs (miR-199a and miR-376). We found no significant differences of these miRNAs at the expression level, with either miR-34 or ASrc overexpression (data not shown). These data support the specificity of the effect of miR-34 and exclude a possible pleotrophic effect of miR-34. In other experiments, we performed western blot analysis of known miR-34a targets, such as Axl, c-Myc and β-Catenin (Figure S8) [25], [28], [29]. As expected, we observed a clear downregulation of all proteins investigated upon miR-34 overexpression. Interestingly, we also observed an induction of Axl protein amounts after overexpression of ASrc, which is know to be transcriptionally regulated by AP-1 family members [30]. Finally, we observed a significant downregulation of Axl protein expression by miR-34a, which was not affected by the additional overxpression of ASrc. Based on these data, we conclude that the effect of miR-34a is due to a direct inactivation of AP-1, leading to downregulation of miR-21 expression.

### miR-34a Inhibits A-Src-induced Migration and Invasion

**Figure 6:**
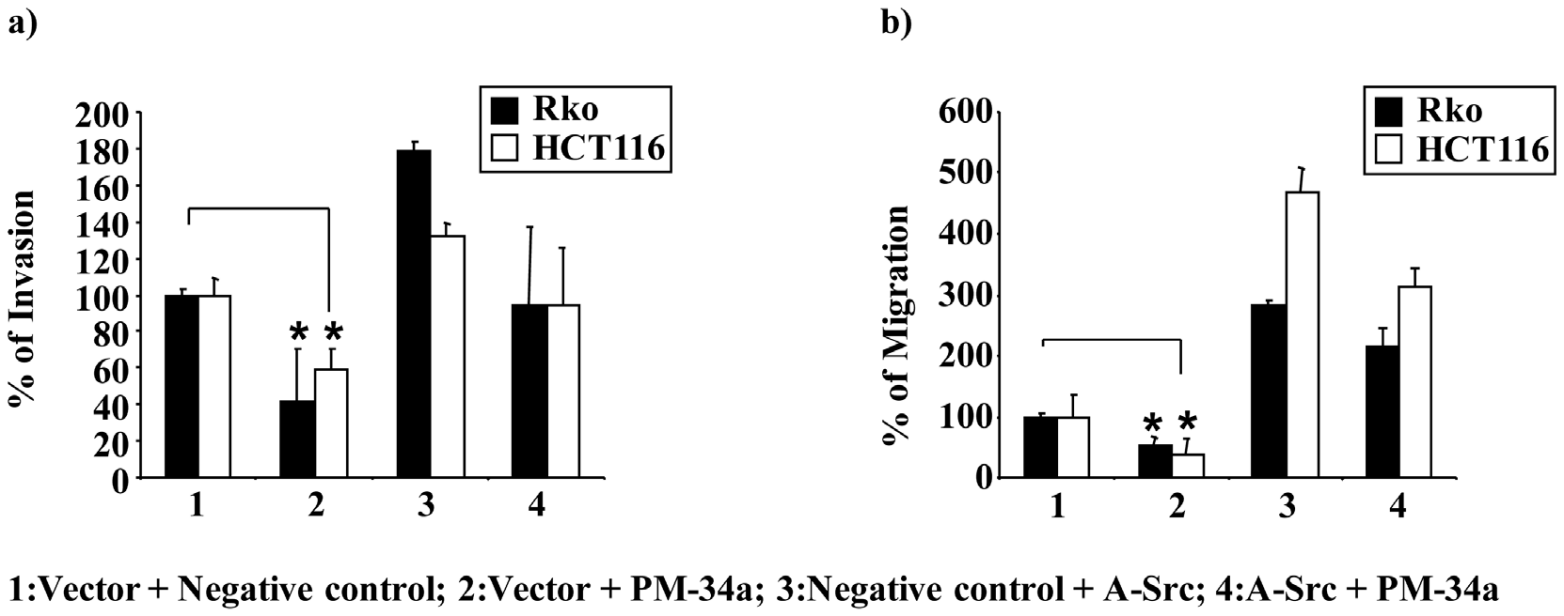
miR-34a inhibits migration and invasion. (**a**) PM-34a inhibits migration *in vitro*. Rko and HCT-116 cells were transfected with a constitutively active Src expression construct (A-Src), empty vector (Vector), PM-34a or negative control (NC) as indicated. After 48 h, the cells were counted and equal numbers of cells were seeded on top of transwell plates with serum free medium and 0.1% BSA. After 14 h, the migrated cells were measured as described in Materials and Methods. Data are represented as the percentage of migrated cells, as mean±s.d. of triplicates (*p≤0.05). (**b**) PM-34a inhibits invasion *in vitro*. Rko and HCT-116 cells were transfected with a constitutively active Src expression construct (A-Src), empty vector (Vector), PM-34a or negative control (NC) as indicated. After 48 h, the cells were counted and equal numbers of cells were seeded in serum free medium and 0.1% BSA on top of transwell chambers coated with Matrigel. After 14 h, invaded cells were measured as described in Materials and Methods. Data are represented as the percentage of invaded cells, as mean±s.d. of triplicates (*p≤0.05).

Several publications have demonstrated a function for CD24, Src, Pdcd4, miR-21 and miR-34a in migration, invasion and metastasis [7], [14], [20], [23], [25], [31]. To determine whether miR-34a suppresses A-Src-induced tumor progression, we performed migration and invasion assays. Transfection of Rko and HCT-116 cells with PM-34a significantly inhibited A-Src-dependent migration and invasion (Figure 6a,b). These data allow us to conclude that CD24/Src-mediated tumor progression can be inhibited by miR-34a.

### CD24 and Src are Coexpressed in Resected Colorectal Tumors

**Figure 7:**
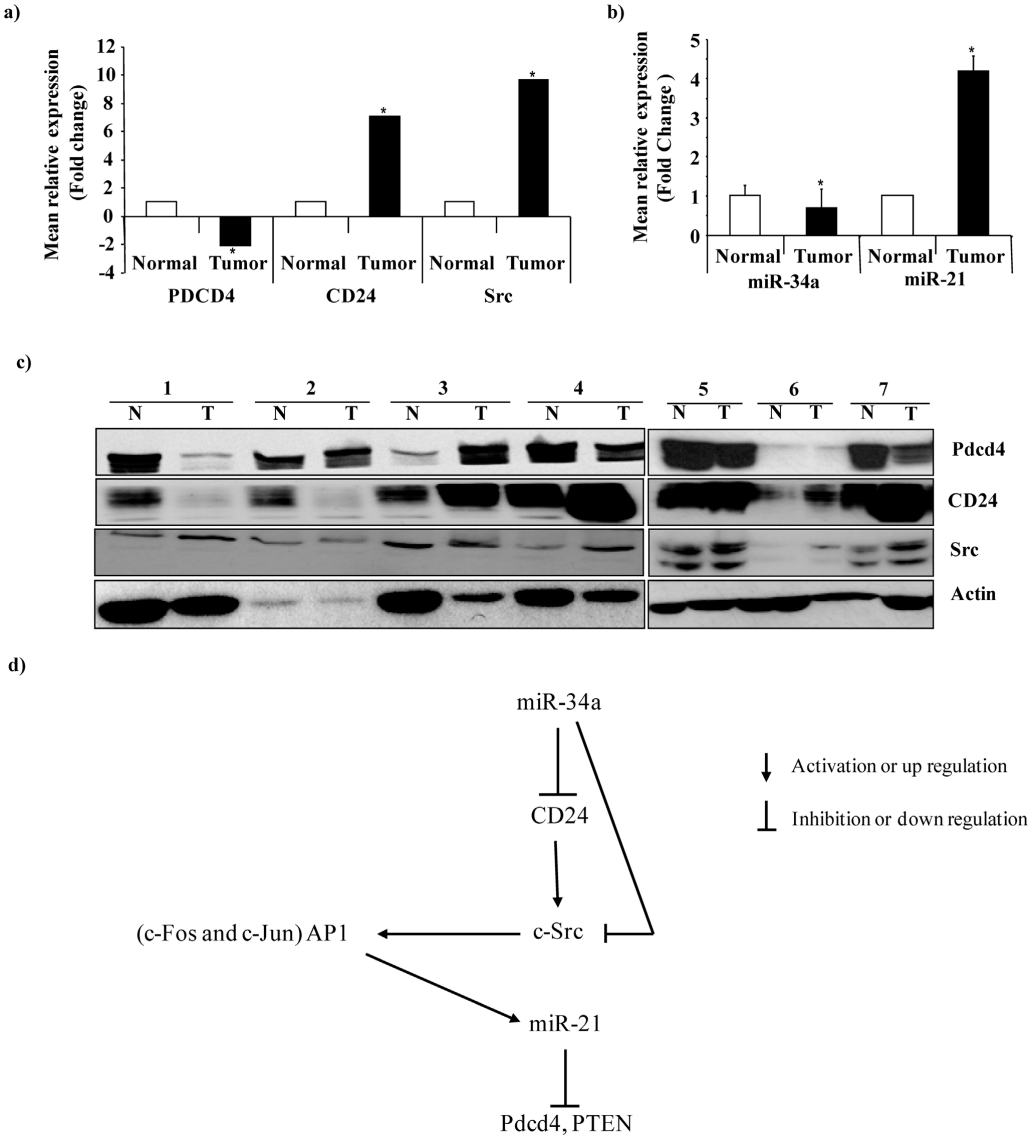
Endogenous expression of Pdcd4, CD24, Src, miR-21 and miR-34a in resected colorectal tissues. (**a**) Western blot analysis was performed for Pdcd4, CD24 and Src in colorectal tumors (Tumor) and corresponding normal tissues (Normal) taken from a series of 26 patients. β-Actin served as internal control. Relative mean protein amounts (Fold change comparative to normal tissue expression) of Pdcd4, CD24 and Src obtained by densitometry analysis are represented as bar graphs. Specific Pdcd4, CD24 or Src band intersities were normalized with β-actin. Pdcd4 was downregulated, CD24 and Src were upregulated significantly in the tumor tissues (p = 0.003, p = 0.05 and p = 0.001, respectively) (**b**) Real-time PCR results of miR-21 and miR-34a in the same colorectal tumor (Tumor) and normal tissue (Normal) samples. Mean relative expression (fold change compared to expression in normal tissue) of miR-21 and miR-34a is represented as bar graphs. miR-21 was upregulated and miR-34a was downregulated significantly in the tumor tissues. (p = 0.002, p = 0.05, respectively) (**c**) Lysates from 7 representative normal tissue (N) and colorectal tumor (T) samples were subjected to Western blotting and probed for the expression of Pdcd4, CD24 and Src and represented. β-Actin served as a loading control (**d**) Schematic representation of the functional network between CD24, Src, AP-1, miR-21, Pdcd4 and miR-34a.

To determine the *in vivo* relevance of the mechanisms identified, 26 resected colorectal carcinoma tissues of patients and corresponding normal mucosae were investigated for endogenous expression of Pdcd4, CD24, Src, miR-34a and miR-21. As shown in Figure 7, we observed that Pdcd4 and miR-34a were significantly downregulated, whereas CD24, Src, and miR-21 were significantly upregulated in the tumor tissues as compared to the respective normal tissues (p = 0.003; p = 0.05; p = 0.001; p = 0.05 and p = 0.002; respectively). Furthermore, CD24 expression positively and significantly correlated with Src gene expression in the resected tumor tissues (p = 0.001), and Src expression showed a trend towards negative association with miR-34a expression. Additionally, miR-34a expression correlated significantly with pT stage, demonstrating a significant inversive correlation (Correlation Coefficient −0.494; p = 0.023), supporting the relevance of our finding.

## Discussion

In this study we have identified a novel molecular network that regulates tumor progression, in which miRNAs play a pivotal role in determining the expression of the tumor suppressor genes Pdcd4 and PTEN (Figure 7d). Specifically, miRNA-34a suppresses expression of CD24 and Src, thereby diminishing the tumor progression-associated functions of these genes, which results in reduced expression of the oncomir miR-21, and thus relieves miR21-mediated repression of Pdcd4 and PTEN expression. In the absence of miRNA-34a, expression of CD24 activates Src, which in turn induces AP-1 family members and stimulates expression of miR-21. Expression of Pdcd4 and PTEN is subsequently suppressed by miR-21. These data suggest that the balance between oncogenically-acting and tumor-suppressing miRNAs can determine the course of tumor progression, and cast new light on how CD24 and Src act to promote tumor growth and metastasis.

Although we have focused here on expression of miR21, Pdcd4 and PTEN as endpoints of the molecular regulatory network we describe, it is likely that effects of the network extend beyond these genes. Src activates AP-1 family members through the MAPK pathway [18], [31], which in turn induces a number of tumor- and metastasis-promoting molecules including u-PAR [31]. Furthermore, Src regulates a variety of other signal transduction pathways, including the PI3K/Akt, STAT and FAK pathways, and regulates Rho family GTPases, resulting in the regulation of a variety of cellular processes including cytoskeletal architecture remodelling, motility, invasion, cell adhesion and the epithelial-mesenchymal transition [32]. Moreover, we show that CD24/Src-dependent upregulation of miR-21 expression is mediated through the AP-1 family members c-Jun and c-Fos, confirming the importance of AP-1 in the regulation of miR-21 expression as described previously [22], [23]. Importantly, miR-21 is a prognostic marker, and the only microRNA upregulated in all major solid tumor types screened so far [33], [34]. Loss and gain of function studies in different types of cancer types have established a role for miR-21 in cell proliferation, inhibition of apoptosis, migration, invasion and distant metastasis [20], [21], [35], [36], [37], and a number of cancer-relevant miR-21 target genes in addition to Pdcd4 and PTEN have been identified [38], [39], [40]. Thus the regulatory network we describe is likely to affect expression of many Src-regulated and miR-21 target genes.

Although activating mutations in the Src gene are rarely found in tumors [15], Src is often overexpressed in tumors, but high protein levels do not necessarily correlate with Src kinase activity [17]. For a number of tumor types including lung, breast and colon cancers, there is an association between Src activity and poor clinical prognosis [16], [41], [42]. We have previously shown that expression of CD24 is a mechanism that can activate Src kinase activity in the tumor context [14], consistent with the observation that the Ras/ERK/MAPK pathway that is activated by Src [43] is downregulated as a consequence of targeting of CD24 [27]. Here we show a further facet of the tumor-relevant regulation of Src, namely that the tumor suppressor miRNA-34a inhibits its expression. Loss of miRNA-34a expression during tumor progression is therefore likely to contribute to the enhanced levels of Src protein observed during the progression of many types of tumor.

The tumor suppressor function of miR-34a is reflected in its epigenetic silencing during tumor progression [44], its positive regulation by p53 [24], and its ability to target and suppress expression of a number of oncogenes and other cancer relevant genes, including Axl, c-Met, CD44, MYCN, Notch1, Jagged 1, the Notch ligand Delta-like1, and the cell cycle regulators CCND1 and CDK6 [25], [45], [46], [47], [48], [49]. Interestingly, other miR-34a target genes such as the deacetylase SIRT1 and the transcription factor YY1 both suppress p53 activity/expression [50], [51], suggesting that a positive feedback loops exists in which p53 induces miR-34a, which in turn suppresses the p53 inhibitors SIRT1 and YY1. Our study casts further light on the tumor suppressor function of miR-34a. We demonstrate that it can suppress the tumor progression genes Src and CD24. Furthermore, we show it can indirectly diminish expression of the oncomir miR-21 by targeting CD24 and Src expression post-transcriptionally. Post-transcriptional regulation of Src by miR-203 and miR-205, two other miRNAs with tumor suppressor functions, has also been reported in bladder and renal cancer [52], [53]. Thus it is likely that miR-203 and miR-205 also diminish miR-21 expression through their suppression of Src.

The findings we present here are relevant to human cancer and have potential therapeutic application. Although we only used a small patient cohort in this study, nevertheless we saw a clear correlation between downregulation of Pdcd4 and miR-34a and enhanced expression of miR-21, CD24 and Src in the resected colorectal tumor samples compared to normal tissue. Moreover, CD24 and Src expression positively correlated with each other, and miR-34a expression was negatively associated with Src in the tumor tissues. Further work will confirm these findings in a larger series of patients. Nevertheless, our findings suggest that reversing the epigenetic silencing of miR-34a could be therapeutically beneficial for colorectal cancer patients. We note with interest that treatment of prostate cancer patients with BioResponse 3,3′- Diindolylmethane (BR-DIM) resulted in re-expression of miR-34a due to reversal of the methylation-induced silencing of the miR-34a promoter, and suppressed the expression of miR-34a target genes including the androgen receptor and Notch-1 [54]. These findings demonstrate the feasibility of reversing epigenetic silencing of miR-34a for therapeutic purposes in the context of cancer.

## Supporting Information

Figure S1 **Endogenous expression of CD24, Src, miR-21, miR-34a and Pdcd4 in a panel of human cancer cell lines.** (a) Relative expression of CD24, Src and Pdcd4 in a panel of colorectal cell lines and in the MDA-MB-231 breast cancer cell line was evaluated by Real-Time PCR. β-Actin served as an internal control. (b) Western blot analysis of CD24, phosphor-Src (p-Src), Src and Pdcd4 in a panel of colorectal cell lines and in the MDA-MB-231 breast cancer cell line. β-Actin served as an internal control. (c) Relative expression of miR-34a and miR-21 in a panel of colorectal cell lines and in the MDA-MB-231 breast cancer cell line was evaluated by Real-Time PCR. RNUB6 served as an internal control.
(TIF)

Figure S2 **Expression of CD24 and Src.** (a) Rko and HCT-116 cells were transfected either with vector control or a CD24 expression construct. HT-29 and Geo cells were transfected with negative control siRNA or with siRNA against CD24. After 48 h, expression levels were determined by Real-Time PCR. (b) Rko and HCT-116 cells were transfected with either vector control or a constitutively active Src expression construct (A-Src). HT-29 and Geo cells were transfected with either negative control siRNA or with siRNA against Src. After 48 h, expression levels were determined by Real-Time PCR. Each bar represents the mean value of three biological replicates.
(TIF)

Figure S3 **CD24-dependent regulation of c-Jun, c-Fos and Pdcd4 is mediated through Src.** c-Fos, c-Jun and Pdcd4 mRNA expression was evaluated by RT-PCR 48 h post transfection of (Top row) or AP-1 4X Luc luciferase activity (Bottom row) Rko and Geo cells with either empty vector (Vector), a CD24 expression construct (CD24), Negative control siRNA (NC) or with an siRNA against Src as indicated. Each bar represents the mean value of three biological replicates (*p≤0.05).
(TIF)

Figure S4 **miR-34a target sites within the CD24- and Src-3′-UTR.** The location of the putative miR-34a target sites in CD24 and Src transcripts (WT) and their mutated variants (MT) are shown with underlined and bold letters, respectively.
(TIF)

Figure S5 **Dose dependent expression of miR-34a.** Luciferase assays of the CD24- and Src-3′-UTR reporter constructs transfected into Rko and HCT116 cells together with either control-miRNA (NC) or increased concentrations of PM-34a as mentioned. Percent luciferase activity was calculated either with the CD24- or Src-3′-UTR or control-miR samples set as 100%. The data are presented as the mean ± S.D. Each bar represents the mean value of three technical replicates (NC: Negative control).
(TIF)

Figure S6 **Expression of CD24 and Src mRNA upon transfection with PM-34a.** Geo cells were transfected either with control miRNA or with PM-34a. After 48 h, expression of CD24 and Src mRNA was determined by Real-Time PCR. Data is represented as the mean of three technical replicates. (NC: Negative control)
(TIF)

Figure S7 **Src-induced c-Fos, c-Jun and Pdcd4 expression is antagonised by PM-34a.** c-Fos, c-Jun and Pdcd4 mRNA expression levels were evaluated by RT-PCR 48 h post transfection (Top row) or AP-1 4X Luc luciferase activity (Bottom row) of Rko and Geo cell lines, with either empty vector (Vector), a constitutively active Src expression construct (A-Src), PM-34a or a negative control (NC) as indicated. Each bar represents the mean value of three biological replicates (*p≤0.05).
(TIF)

Figure S8 **miR-34a target molecules.** Western blot analysis of known miR-34a target molecules Axl, c-Myc and β-Catenin was performed 48 h post transfection. Rko cells were transfected with a constitutively active Src expression construct (A-Src), empty vector (Vector), PM-34a or scrambled siRNA control (Scrambled) as indicated. β-Actin served as an internal control.
(TIF)

Table S1 **Oligonucleotides used in this study.**
(DOC)
