# Correction: CD24 Induces Expression of the Oncomir miR-21 via Src, and CD24 and Src Are Both Post-Transcriptionally Downregulated by the Tumor Suppressor miR-34a

**DOI:** 10.1371/journal.pone.0118441

**Published:** 2015-02-06

**Authors:** The PLOS ONE Staff

The following information is missing from the Funding section: GM, JL, JS and HA were supported by Wilhelm Sander Foundation, Munich, Germany (2012.036.1). Please refer to the complete Funding section here.

SM was supported by a doctoral stipendium awarded to JPS and HA by the Medical Faculty Mannheim, University Heidelberg, under the auspices of the Forschungsschwerpunkt Oncologie initiative. GM was supported by the Stiftung fur krebs- und Scharlachforschung Mannheim, from the University of Heidelberg. JPS gratefully acknowledges funding from the European Union under the auspices of the FP7 collaborative project TuMIC, contract no. HEALTH-F2-2008-201662. HA was supported by the Alfried Krupp von Bohlen und Halbach Foundation (Award for Young Full Professors), Essen, Hella-Bühler-Foundation, Heidelberg, the Hector Foundation, Weinheim, the FRONTIER Excellence Initiative of the University of Heidelberg, the BMBF, Bonn, the Walter Schulz Foundation, Munich, Deutsche Krebshilfe, Bonn, Germany, and the German-Israeli Cooperation (DKFZ-MOST). GM, JL, JS and HA were supported by Wilhelm Sander Foundation, Munich, Germany (2012.036.1). The funders had no role in study design, data collection and analysis, decision to publish, or preparation of the manuscript.
