# Supplementary figures and images for: CD24 Induces Expression of the Oncomir miR-21 via Src, and CD24 and Src Are Both Post-Transcriptionally Downregulated by the Tumor Suppressor miR-34a
Source: PLoS One. 2013 Mar 22;8(3):e59563. doi: 10.1371/journal.pone.0059563 (PMC3606220; doi:10.1371/journal.pone.0059563)

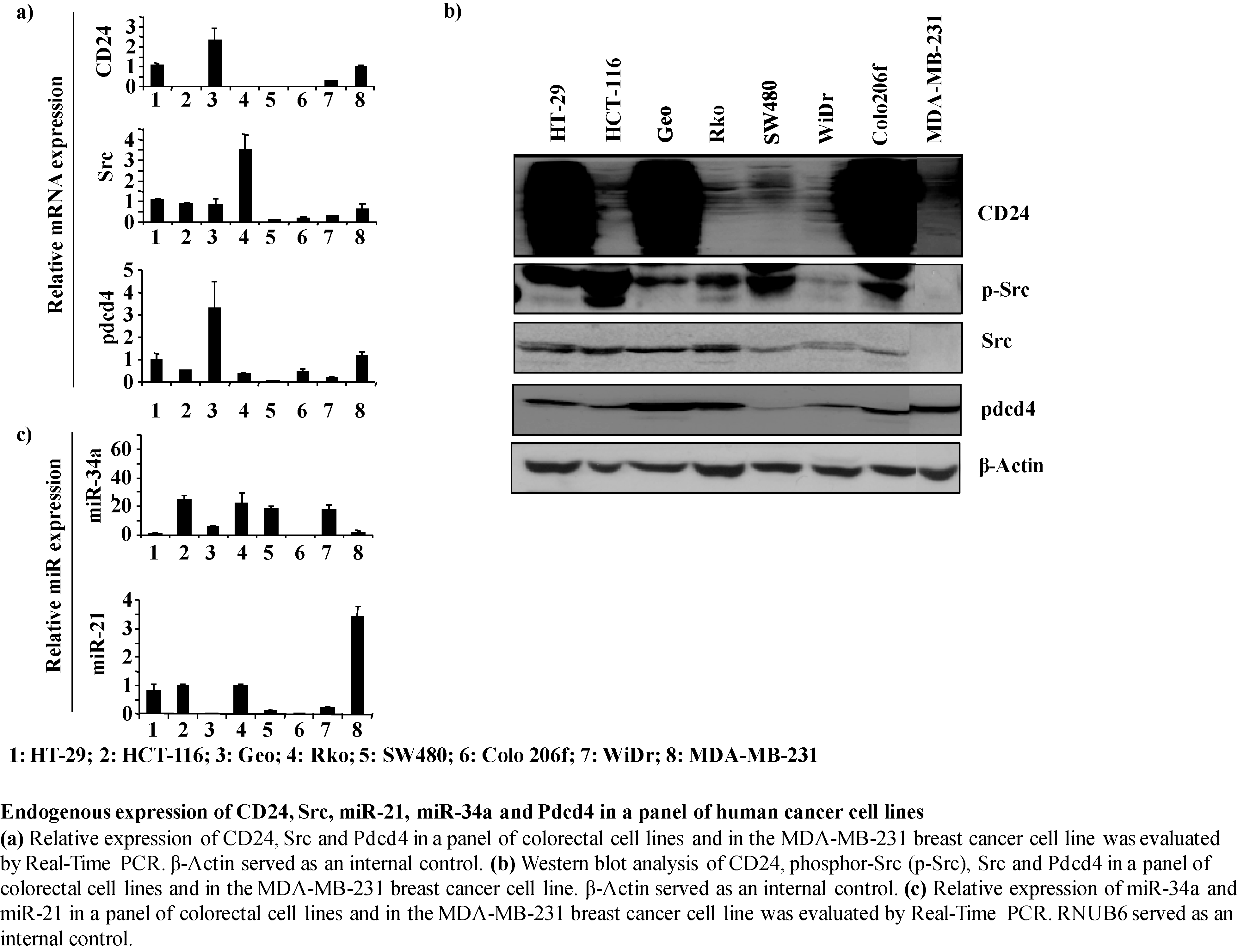

Supplement: Figure S1 — Endogenous expression of CD24, Src, miR-21, miR-34a and Pdcd4 in a panel of human cancer cell lines. (a) Relative expression of CD24, Src and Pdcd4 in a panel of colorectal cell lines and in the MDA-MB-231 breast cancer cell line was evaluated by Real-Time PCR. β-Actin served as an internal control. (b) Western blot analysis of CD24, phosphor-Src (p-Src), Src and Pdcd4 in a panel of colorectal cell lines and in the MDA-MB-231 breast cancer cell line. β-Actin served as an internal control. (c) Relative expression of miR-34a and miR-21 in a panel of colorectal cell lines and in the MDA-MB-231 breast cancer cell line was evaluated by Real-Time PCR. RNUB6 served as an internal control. (TIF) [file pone.0059563.s001.tif]

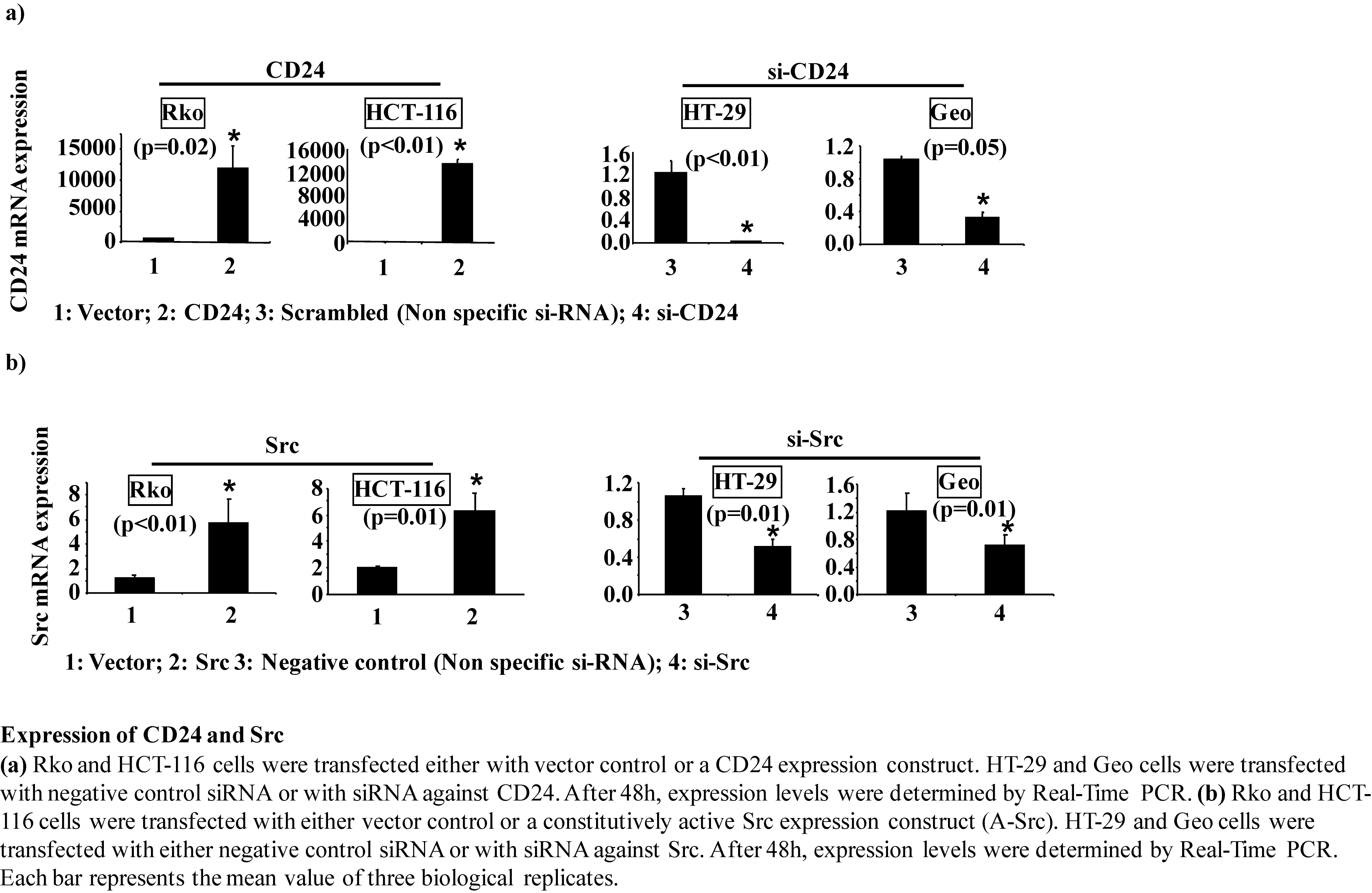

Supplement: Figure S2 — Expression of CD24 and Src. (a) Rko and HCT-116 cells were transfected either with vector control or a CD24 expression construct. HT-29 and Geo cells were transfected with negative control siRNA or with siRNA against CD24. After 48 h, expression levels were determined by Real-Time PCR. (b) Rko and HCT-116 cells were transfected with either vector control or a constitutively active Src expression construct (A-Src). HT-29 and Geo cells were transfected with either negative control siRNA or with siRNA against Src. After 48 h, expression levels were determined by Real-Time PCR. Each bar represents the mean value of three biological replicates. (TIF) [file pone.0059563.s002.tif]

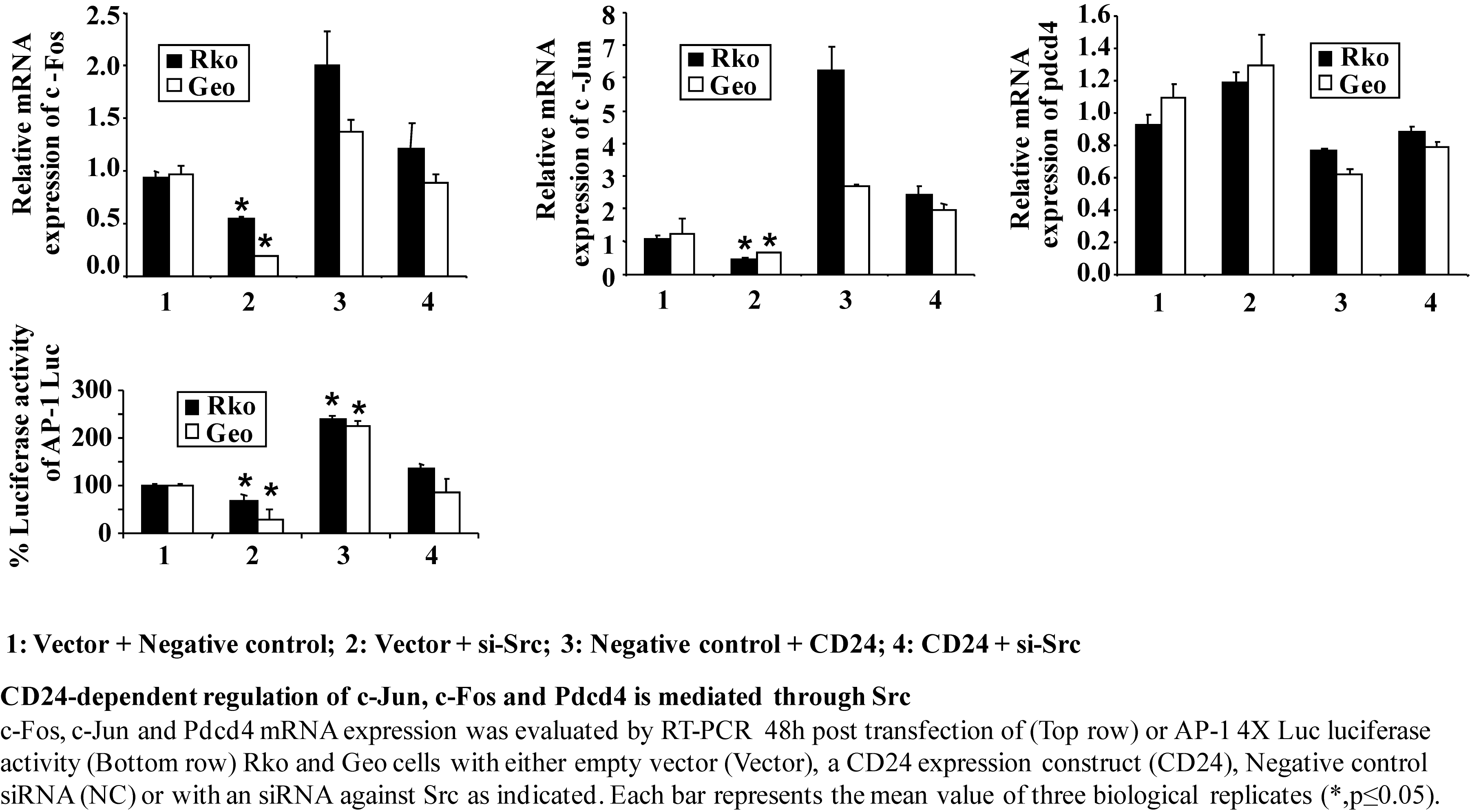

Supplement: Figure S3 — CD24-dependent regulation of c-Jun, c-Fos and Pdcd4 is mediated through Src. c-Fos, c-Jun and Pdcd4 mRNA expression was evaluated by RT-PCR 48 h post transfection of (Top row) or AP-1 4X Luc luciferase activity (Bottom row) Rko and Geo cells with either empty vector (Vector), a CD24 expression construct (CD24), Negative control siRNA (NC) or with an siRNA against Src as indicated. Each bar represents the mean value of three biological replicates (*p≤0.05). (TIF) [file pone.0059563.s003.tif]

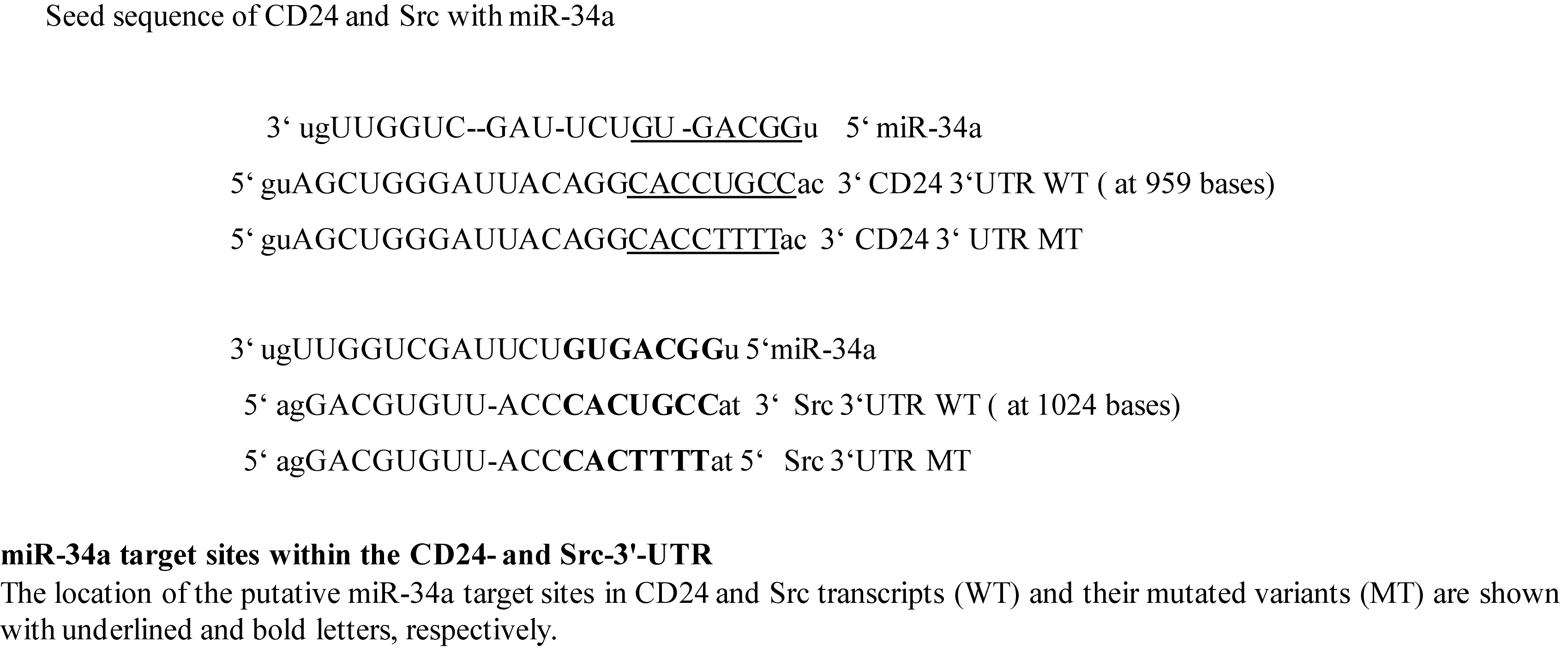

Supplement: Figure S4 — miR-34a target sites within the CD24- and Src-3′-UTR. The location of the putative miR-34a target sites in CD24 and Src transcripts (WT) and their mutated variants (MT) are shown with underlined and bold letters, respectively. (TIF) [file pone.0059563.s004.tif]

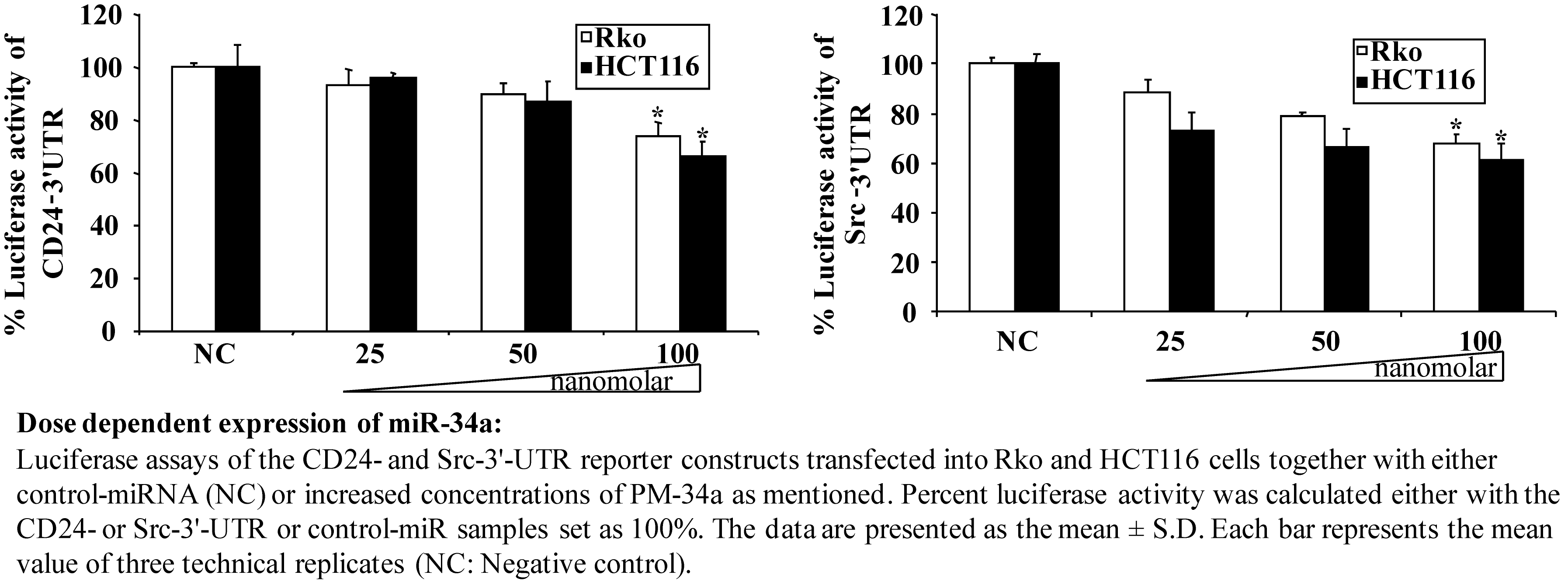

Supplement: Figure S5 — Dose dependent expression of miR-34a. Luciferase assays of the CD24- and Src-3′-UTR reporter constructs transfected into Rko and HCT116 cells together with either control-miRNA (NC) or increased concentrations of PM-34a as mentioned. Percent luciferase activity was calculated either with the CD24- or Src-3′-UTR or control-miR samples set as 100%. The data are presented as the mean ± S.D. Each bar represents the mean value of three technical replicates (NC: Negative control). (TIF) [file pone.0059563.s005.tif]

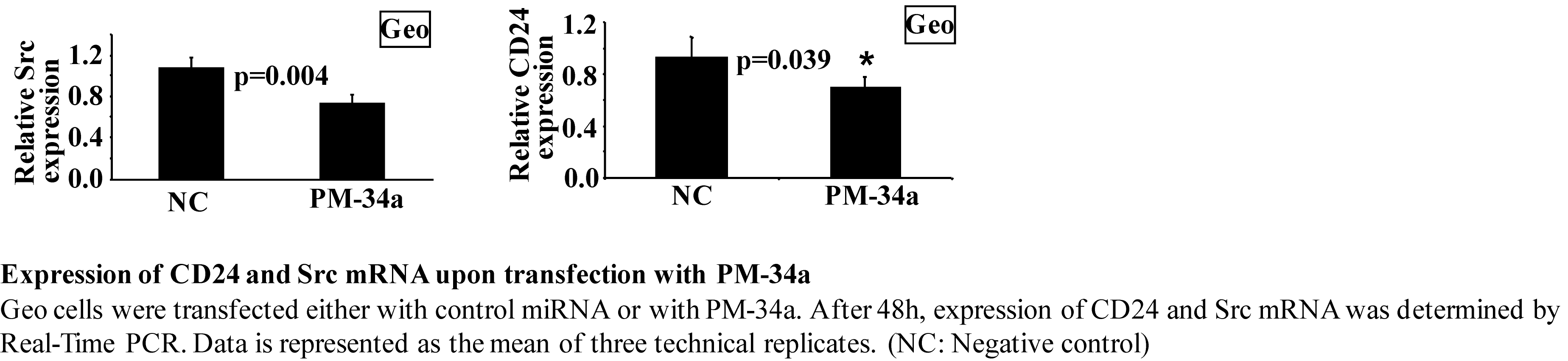

Supplement: Figure S6 — Expression of CD24 and Src mRNA upon transfection with PM-34a. Geo cells were transfected either with control miRNA or with PM-34a. After 48 h, expression of CD24 and Src mRNA was determined by Real-Time PCR. Data is represented as the mean of three technical replicates. (NC: Negative control) (TIF) [file pone.0059563.s006.tif]

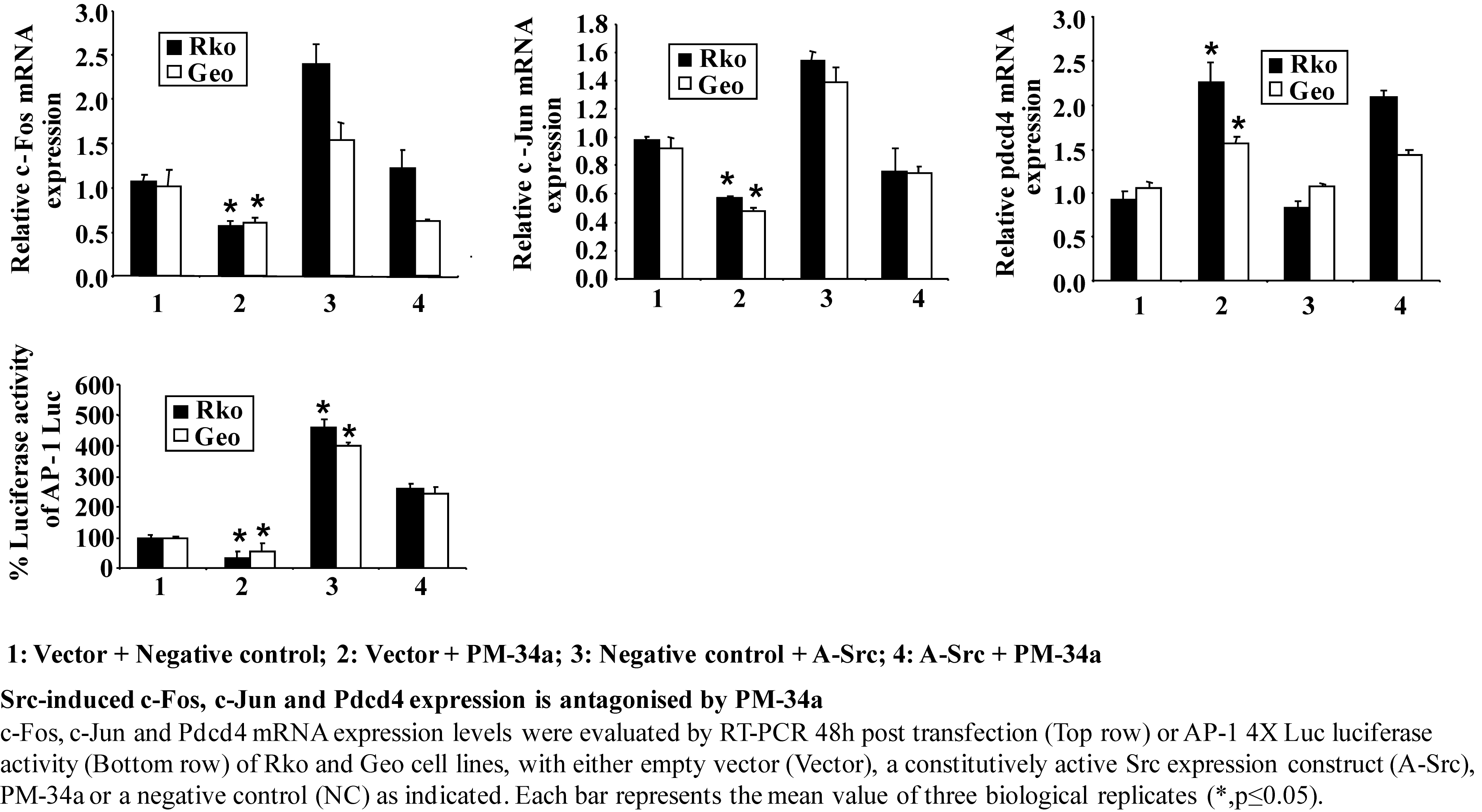

Supplement: Figure S7 — Src-induced c-Fos, c-Jun and Pdcd4 expression is antagonised by PM-34a. c-Fos, c-Jun and Pdcd4 mRNA expression levels were evaluated by RT-PCR 48 h post transfection (Top row) or AP-1 4X Luc luciferase activity (Bottom row) of Rko and Geo cell lines, with either empty vector (Vector), a constitutively active Src expression construct (A-Src), PM-34a or a negative control (NC) as indicated. Each bar represents the mean value of three biological replicates (*p≤0.05). (TIF) [file pone.0059563.s007.tif]

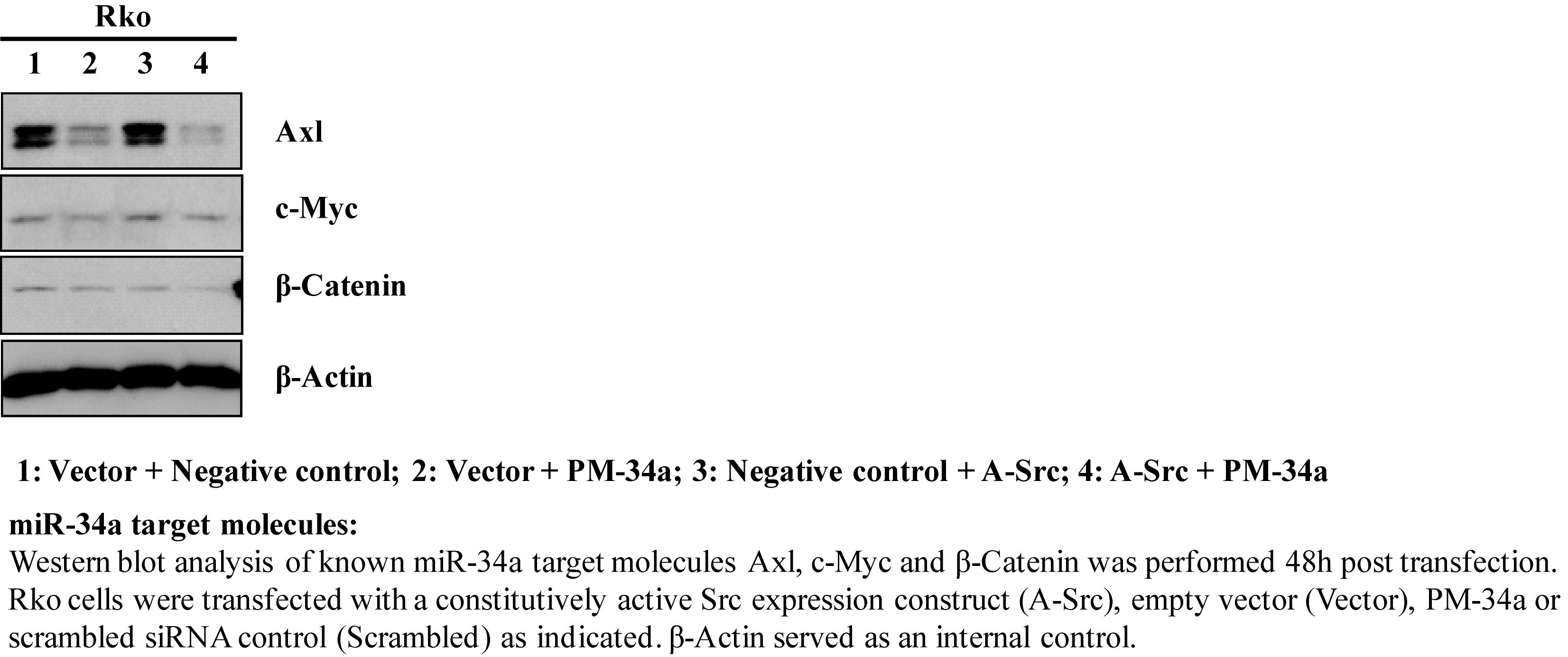

Supplement: Figure S8 — miR-34a target molecules. Western blot analysis of known miR-34a target molecules Axl, c-Myc and β-Catenin was performed 48 h post transfection. Rko cells were transfected with a constitutively active Src expression construct (A-Src), empty vector (Vector), PM-34a or scrambled siRNA control (Scrambled) as indicated. β-Actin served as an internal control. (TIF) [file pone.0059563.s008.tif]
