# Supplementary material for: CD24 Induces Expression of the Oncomir miR-21 via Src, and CD24 and Src Are Both Post-Transcriptionally Downregulated by the Tumor Suppressor miR-34a
Source: PLoS One. 2013 Mar 22;8(3):e59563. doi: 10.1371/journal.pone.0059563 (PMC3606220; doi:10.1371/journal.pone.0059563)
Supplement: Table S1 — Oligonucleotides used in this study. (DOC) [file pone.0059563.s009.doc]

**Supplementary Table 1**

**Oligonucleotides used in this study**

| **S.No** | **Gene Name** | **Sequence (5’ --- 3’)** |
| --- | --- | --- |
|  | **CD24 cloning** | |
| 1 | CD24_Const_For | AAGGATCCgaggggacatgggcagag |
| 2 | CD24_Const_Rev | AACTCGAGttaagagtagagatgcagaagagaga |
|  | **CD24 and Src 3’UTR Cloning** | |
| 3 | CD24_UTR_For | TAAAGCTTatgtggcaaggaaaaacagg |
| 4 | CD24_UTR_Rev | TGAAGCTTtcgatctgtttgttcccatgt |
| 5 | Src_UTR_For | TTTCTAGAcctgctgttggtcctctctc |
| 6 | Src_UTR_Rev | GATCTAGAaagtctgggccttggaattt |
|  | **CD24 and Src 3’UTR mutations** | |
| 7 | CD24_UTR_M_For | gctgggattacaggcaccttttaccatgggctaatgtaaag |
| 8 | CD24_UTR_M_Rev | ctttacattagcccatggtaaaaggtgcctgtaatcccagc |
| 9 | Src_UTR_M_For | gaaagaggacgtgttacccacttttatgcaccaggactggctgtg |
| 10 | Src_UTR_M_Rev | cacagccagtcctggtgcataaaagtgggtaacacgtcctctttc |
|  | **RT_PCR primers** | |
| 11 | CD24_RTPCR_For | tccaaggcacccagcatcctgctaga |
| 12 | CD24_RTPCR_Rev | tagaagacgtttcttggcctgagtct |
| 13 | Src_RTPCR_For | ggcggcttctacatcacct |
| 14 | Src_RTPCR_Rev | agggatctcccaggcatc |
| 15 | β-Actin_For | tccctggagaagagctacg |
| 16 | β-Actin_Rev | gtagtttcgtggatgccaca |
| 17 | c-Jun_For | acgcaaacctcagcaacttc |
| 18 | c-Jun_Rev | cactgtctgaggctcctcct |
| 19 | c-Fos_For | agaatccgaagggaaaggaa |
| 20 | c-Fos_Rev | cttctccttcagcaggttgg |
